# Epidemiological characteristics of inhaled allergens influenced by plum rains and diagnostic value of neutrophil to lymphocyte ratio in atopic dermatitis patients

**DOI:** 10.1097/MD.0000000000044866

**Published:** 2025-10-10

**Authors:** Qingyun Qu, Qian Sun

**Affiliations:** a Department of Clinical Laboratory, Xiangya Hospital, Central South University, Changsha, Hunan Province, China; b Department of Clinical Laboratory, National Clinical Research Center for Geriatric Disorders, Xiangya Hospital, Central South University, Changsha, Hunan Province, China.

**Keywords:** atopic dermatitis, inhaled allergen, mold, neutrophil to lymphocyte ratio, plum rains

## Abstract

The aim of this study was to explore the epidemiological characteristics of inhaled allergens influenced by the plum rains climate in Hunan and the diagnostic value of blood routine inflammation indicators for atopic dermatitis. The clinical data of 3544 patients with atopic dermatitis from the Xiangya Hospital of Central South University from 2020 to 2022 were retrospectively analyzed. Meanwhile, the meteorological data of Hunan during the plum rains period from the China Meteorological Network were collected, and the statistical indices were analyzed using SPSS26 statistical software. The results showed that the inhaled allergens in Hunan were mainly dust mites (20.12%) and house dust (13.09%). The positive rate of males was significantly higher than that of females (*P* < .001), and the positive rate of children aged 1 to 11 years was the highest (51.3%). Furthermore, the positive rate of specific allergens was 31.74% in the plum rains season, which was significantly higher than that of 24.30% in the non-plum rains season (*P* < .001), and the positive rate of mold specific allergens was significantly higher in the plum rains season than in the non-plum rains season (*P* = .016). Significant differences in neutrophil to lymphocyte ratio (NLR), lymphocyte to monocyte ratio (LMR) and platelet to lymphocyte ratio (PLR) were observed between the experimental and control groups. ROC analysis showed that the sensitivity and specificity of the NLR in the diagnosis of atopic dermatitis were 71.6% and 62.3%, respectively. Our findings suggest that there are gender and age differences in the prevalence of inhaled allergens in atopic dermatitis patients in Hunan, and the positive rate of specific allergens was positively correlated with plum rains climate, especially mold allergens. The NLR should be used as a potential new marker for the diagnosis of atopic dermatitis.

## 1. Introduction

In recent years, with changes in the human living environment and lifestyle, the incidence of allergic diseases in the population has shown a rapid increase. The World Health Organization has pointed out that allergic diseases have affected or are affecting 30 to 40% of the global population.^[1]^ The prevalence of allergic diseases in China has shown an increasing trend. A study on Chinese adult lung health published in the Lancet showed that there were 45.7 million asthma patients were aged 20 years and above in China, and the prevalence rate of adult asthma reached 4.2%.^[2]^ Common inhaled allergens, such as dust mites, plant pollen and animal fur, can lead to allergic rhinitis, allergic asthma, allergic urticaria, and even anaphylactic shock.^[3]^ Atopic dermatitis, a chronic, relapsing, inflammatory allergic skin disease, is characterized by recurrent chronic eczematoid rash, accompanied by significant skin dryness and pruritus. The prevalence of childhood atopic dermatitis ranged from 15 to 30% while adult atopic dermatitis from 2% to 10% in industrialized countries, and the prevalence of atopic dermatitis in China has been increased during the past decade.^[4]^ Atopic dermatitis is often associated with other allergic conditions, and its repeated attacks and prolonged treatment seriously affect the physical and mental health of patients, bringing a huge burden to patients. Although the exact pathogenesis of atopic dermatitis is still unclear, current studies believe that immune inflammation is an important link in the pathogenesis of this disease, and some studies have reported that inflammatory indicators are an important factor mediating the pathogenesis of atopic dermatitis.^[5]^ Neutrophil to lymphocyte ratio (NLR) and platelet to lymphocyte ratio (PLR), which have been widely studied, are nonspecific markers that can reflect the degree of systemic inflammation and immune status,^[6,7]^ so NLR and PLR may be of value for early clinical judgment of atopic dermatitis.

Environmental factors play an important role in the occurrence and development of allergic diseases, especially for atopic dermatitis. In recent years, a large number of studies have confirmed that environmental factors such as air pollution, climate, and geographical location all participate in and affect the occurrence and development of allergic diseases.^[8,9]^ Allergen isolation is currently considered the most effective, economical, and convenient means of preventing allergic diseases. Therefore, it is necessary to consider the differences in climate and environment in allergen prevention. At present, respiratory allergic diseases caused by spring and autumn pollen have attracted more attention,^[10]^ although pollen is not the most important allergen in southern China.^[11]^ Plum rains, refers to the natural climate phenomenon of continuous rains from late June to early July in the middle and lower reaches of the Yangtze River in China. Constant high temperature and high humidity during the plum rain period can easily cause significant environmental changes.^[12]^ However, plum rains, an allergic disease caused by climate change, have received less attention. Therefore, it is important to study the influence of plum rains on the prevalence and distribution of inhaled allergens in Hunan Province. This study aimed to investigate the prevalence of inhaled allergens and the influence of plum rains in Hunan and further analyze the diagnostic value of blood routine inflammation indicators for atopic dermatitis.

## 2. Methods

### 2.1. General information

**Figure 1.**
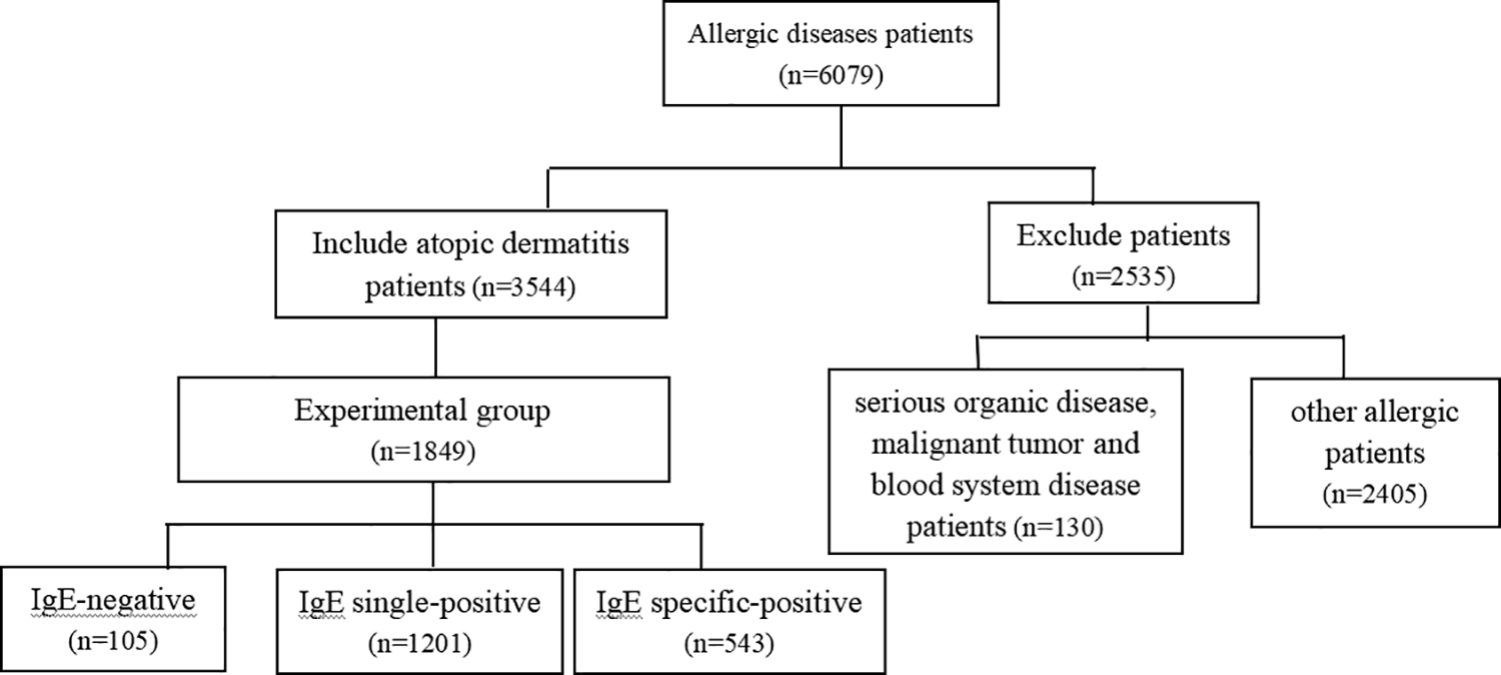
Study flow diagram.

Clinical data of 3544 patients undergoing inhaled allergen-specific IgE antibody screening in the Dermatology Department of Xiangya Hospital, Central South University, from 2020 to 2022 were retrospectively analyzed, including 1321 males and 2223 females, aged from 1 to 86 years old, with an average age of (28.88 ± 17.11) years. They were divided into a group of children (637 cases aged 1–11 years), adolescents (419 cases aged 12–18 years), adults (2341 cases aged 19–60 years), and elderly (147 cases aged > 60 years) according to age. The inclusion criteria were complete clinical data, clinical features, and allergen results for the diagnosis of atopic dermatitis. According to the Chinese guidelines for diagnosis and treatment of atopic dermatitis (2020 edition), the diagnostic criteria for atopic dermatitis in China are as follows: symmetrical eczema with a course of more than 6 months; personal and/or family history of atopy (including eczema, allergic rhinitis, asthma, etc); and elevated serum total IgE and/or positive allergen-specific IgE. The diagnosis can be made if the first item is met, in addition to any one of the 2 or 3 items.^[13]^ Exclusion criteria included serious organic disease, malignant tumor and blood system disease. Among them, 1849 atopic dermatitis patients tested for both routine blood tests and inhaled allergens were further divided into IgE-negative experimental group 1 (105 cases), IgE single-positive experimental group 2 (1201 cases) and IgE specific- positive experimental group 3 (543 cases) according to the allergens to further analyze the value of inflammation indicators in the diagnosis of atopic dermatitis. The control group included 424 healthy people who were admitted to the hospital during the same period, and their gender and age were matched with the experimental group, including 170 males and 254 females, aged from 1 to 90 years, with an average age of (22.12 ± 24.27) years. A flowchart is shown in Figure 1. According to the time range of the plum rains season from 2020 to 2022 released by the China Meteorological Administration, the period of plum rains in Hunan Province from 2020 to 2022 is from June 7 to July 14, June 27 to July 9, and June 17 to July 8, respectively. This study was approved by the Clinical Medical Ethics Committee of the Xiangya Hospital, Central South University.

### 2.2. Detection of inhaled allergens

Enzyme-linked immunosorbent assay was used to detect total IgE antibody and 6 mixed common allergens, including dust mites (house dust mites or dust mites), house dust, tree pollen mix (cypress/elm/sycamore/willow/poplar), plant pollen mix (ragweed/mugwort/wormwood), animal dander (cat dander/dog dander) and mold mix (Penicillium/Aspergillus fumigus/Cladus/Rhizopus alternus/Mucor). The allergen detection kit was purchased from the Suzhou HOB Biotech Group Co., Ltd (Suzhou, Jiangsu, China). The test procedure was performed according to the kit instructions, and 0.35 IU/mL was used as the cutoff value between normal and abnormal.

### 2.3. Detection of blood routine inflammation indicators

Blood routine were detected using a UniCel DxH 800 Beckman Coulter blood and body fluid analyzer (Beckman Coulter Co., Ltd., Pasadena), which count the number of different blood cells (white blood cells and platelets) in given volume of blood. We calculated the NLR, lymphocyte to monocyte ratio (LMR), PLR and mean platelet volume to platelet count (MPV/PC) as follows: NLR = neutrophil counts/lymphocyte counts, LMR = lymphocyte counts/monocyte counts, PLR = platelet counts/lymphocyte counts, MPV/PC = mean platelet volume/platelet counts.

### 2.4. Statistical analysis

SPSS software (version 26.0) was used for the statistical analysis of the data. Normally distributed measurement data were expressed as mean ± standard deviation (*x* ± *s*), and comparisons between groups were performed using the *t*-test. Non-normal distribution measurement data were represented by quartiles, and the Mann–Whitney *U* or Kruskal–Wallis tests were used for comparison. Counting data were expressed as cases or percentages, and the χ^2^ test was used for comparison between groups. The area under curve (AUC) was analyzed using receiver operating characteristic (ROC) curve analysis, and the sensitivity and specificity were calculated after determining the cutoff value. *P* < .05 was considered to be statistically significant.

## 3. Results

### 3.1. Positive rate of common allergens

**Figure 2.**
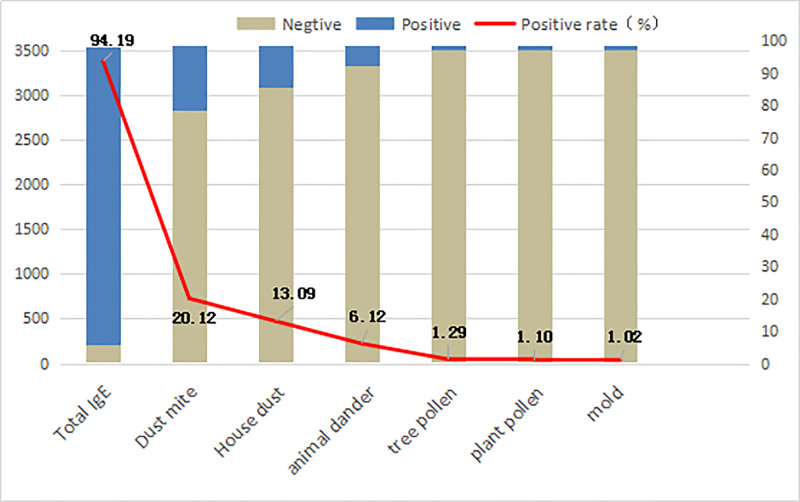
Distribution of inhaled allergens in 3544 cases.

The total IgE positivity rate was 94.19% in 3544 patients with atopic dermatitis. The positive rate for specific allergens was 26.18% (positive for any specific allergen), and the specific allergens were mainly dust mites (20.12%, 713 cases) and house dust (13.09%, 464 cases). Among the 928 patients with positive specific inhalation allergens, 51.94% (482/928) were sensitized to 2 or more allergens, of which 35.88% (333/928) were allergic to dust mites and house dust, as shown in Figure 2.

### 3.2. Analysis of the positive rate of specific allergens between different genders and ages

The positive rate of specific allergens was 30.20% (399/1321 cases) in males and 23.79% (529/2223 cases) in females, and the difference was statistically significant (χ^2^ *=* 17.553, *P* < .001). There was no significant difference in gender between the age groups. The positive rates of specific allergens in the different age groups were 42.07% (268/637 cases) in the children group, 37.95% (159/419 cases) in the youth group, 20.46% (479/2341 cases) in the adult group, and 14.97% (22/147 cases) in the elderly group, and the differences were statistically significant (χ^2^ = 162.762 cases, *P* < .001), as shown in Table 1.

**Table 1:** Positive rates of inhaled allergens in different gender and age groups.

Age	Total	Positive (%)	Male	Positive (%)	Female	Positive (%)	*P*-value
1–11	637	268 (42.07)	330	146 (44.24)	307	122 (39.74)	.250
12–18	419	159 (37.95)	215	87 (40.46)	204	72 (35.29)	.276
19–60	2341	479 (20.46)	701	152 (21.68)	1640	327 (19.94)	.343
>60	147	22 (14.97)	75	14 (18.67)	72	8 (11.11)	.199

### 3.3. Comparison of allergens in plum rains and non-plum rains periods

**Figure 3.**
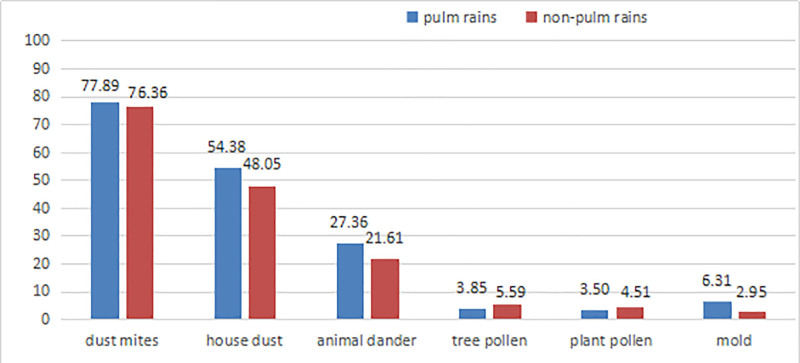
Comparison of allergens in plum rains and non-plum rains season.

The positive rate of specific allergens was 31.74% (285/898 cases) in plum rains periods, which was significantly higher than that of 24.30% (643/2646 cases) in the non-plum rains period (χ^2^ = 19.288, *P* < .001). The positive rates of dust mites, house dust, animal dander and mold in the plum rains period were higher than those in the non-plum rains period, but only the difference in mold between the 2 periods was statistically significant (χ^2^ = 5.827, *P* = .016) (Fig. 3).

### 3.4. Comparison of blood routine inflammatory indicators among different groups

The NLR and PLR in the experimental group were higher than those in the control group, and the LMR was lower than that in the control group, with statistical differences (*P* < .001). The MPV/PC and white blood cell count were not significantly different between the experimental and control groups. There were no statistical differences in all indicators among the 3 groups in the experimental group, as shown in Table 2.

**Table 2:** Comparison of blood routine inflammatory indicators among different groups.

	Control group (n = 424)	Experimental group			*Z*	*P*
		IgE-negative experimental group 1 (n = 105)	IgE single-positive experimental group 2 (n = 1201)	IgE specific-positive experimental group 3 (n = 543)		
NLR	1.08 (0.53–1.77)	1.87 (1.24–2.40)*	1.89 (1.41–2.55)*	1.67 (1.21–2.27)*	236.138	.000
LMR	5.57 (3.80–7.85)	4.50 (3.71–6.00)*	4.25 (3.33–5.60)*	4.50 (3.50–5.80)*	83.79	.000
PLR	93.71 (71.90–126.48)	131 (100.16–176.97)*	128.57 (102.62–159.26)*	123.75 (100–151.36)*	166.964	.000
MPV/PC	0.0353 (0.0257–0.0468)	0.0374 (0.0308–0.0443)	0.0386 (0.0310–0.072)	0.0351 (0.0284–0.0432)	38.455	.000
WBC	6.80 (5.50–8.60)	6.30 (5.45–7.45)	6.60 (5.50–7.90)	6.90 (5.50–8.30)	16.449	.001

NLR = neutrophil to lymphocyte ratio, LMR = lymphocyte to monocyte ratio, PLR = platelet to lymphocyte ratio, MPV/PC = mean platelet volume to platelet count, WBC = white blood cell count.

* Comparison with the control, *P* < .05.

### 3.5. Diagnostic value of blood routine inflammation indicators for atopic dermatitis

**Figure 4.**
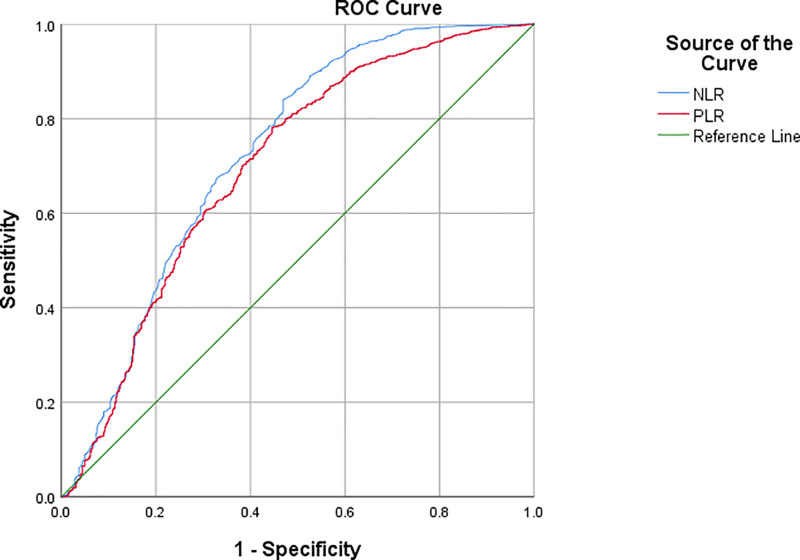
ROC curve of inflammatory indicators in the diagnosis of atopic dermatitis. ROC = receiver operating characteristic.

The ROC results showed that NLR had the highest diagnostic value for atopic dermatitis with an AUC of 0.725. When the cutoff value was 1.405, the sensitivity and specificity were 71.6% and 62.3%, respectively, as shown in Table 3 and Figure 4.

**Table 3:** Diagnostic value of blood routine inflammation indicators for atopic dermatitis.

Indicators	Cutoff	AUC	Sensitivity	Specificity	Jorden index	95% CI
NLR	1.405	0.725	0.716	0.623	0.339	0.695–0.756
PLR	98.67	0.698	0.780	0.554	0.334	0.668–0.728
LMR	6.69	0.637	0.875	0.366	0.241	0.605–0.670

NLR = neutrophil to lymphocyte ratio, LMR = lymphocyte to monocyte ratio, PLR = platelet to lymphocyte ratio.

## 4. Discussion

Atopic dermatitis is a chronic inflammatory skin disease characterized by eczematous dermatitis and itching, and has gradually become a problem affecting global public health and the economy.^[14]^ According to the laboratory characteristics, atopic dermatitis can be divided into endogenous and exogenous type, exogenous type is characterized by high levels of IgE with a personal or familial history of atopic disease and an increase of food or inhaled allergen-specific IgE levels.^[13]^ The prevalence of inhaled allergens has been confirmed to be related to regional differences, living environment, and climatic factors,^[15]^ and is more likely to be recurrent and perennial. Therefore, further understanding of the epidemiological characteristics of inhaled allergens and the influence of seasonal climate change in the Hunan region is important for preventing atopic dermatitis.

House dust mites are the most common allergens that cause allergic diseases worldwide. There are as many as 33 categories of mite allergens in the WHO/IUIS allergen naming database, and a large number of literature have reported that mites are associated with allergic rhinitis, atopic dermatitis, asthma and other diseases.^[16]^ Among the 6 common inhaled allergen allergens in this study, dust mite was the most common inhaled allergen in Hunan, with a positivity rate of 20.12%. Consistent with domestic and foreign literature, mites and their metabolites have become the most important allergens that induce allergic diseases.^[17]^ In this study, the positive rate of house dust-specific allergens reached 16.65%, which is the second most common inhaled allergen in Hunan. However, the positive rate of tree pollen and plant pollen allergens in Hunan is relatively low, which differs from the distribution of common allergens in northern China.^[18]^ Pollen is the second most common allergen in northern China and its content is negatively correlated with precipitation and relative humidity. The Hunan area is located in southern China and experiences heavy rainfall and high relative humidity, which may be more suitable for indoor dust mites and house dust breeding. Dander is a special protein secreted by the salivary and sebaceous glands of cats and dogs and is transferred to the fur by grooming and spread to the surrounding environment. In recent years, with changes in lifestyle, such as raising pets, there have been an increasing number of reports of dander allergies. In this study, the positive rate of animal dander specific allergens rose to third place, and it has gradually become one of the main allergens. Therefore, allergy to animal proteins is an important public health issue that deserves further attention.^[19]^ At the same time, this study found that more than half of the patients were allergic to 2 or more allergens, indicating that allergic diseases are mostly manifested by mixed sensitization to multiple allergens. The positive rate of mixed allergy between the first dust mite and the second house dust reached 35.88%, which may be due to the cross-reaction of produced antibodies after the sensitization of the house dust and mite allergens, which is consistent with the literature reports.^[20]^ Sensitivity to multiple allergens may also be one of the reasons why clinical treatment for allergic diseases is often poor.

The plum rains season is a long period of hot, humid, and rainy weather that often occurs in the middle and lower reaches of the Yangtze River in China. This climate is conducive to the growth and reproduction of pathogenic microorganisms, which can easily lead to allergic diseases such as asthma and rhinitis. There are also literature analyses showing that 9 major cities in the middle and lower reaches of the Yangtze River in China are affected by the plum rains season, among which Changsha in Hunan Province has the highest condensation risk and the greatest difference in humidity change.^[21]^ This study found that the total positive rate of common allergens during the plum rains season was higher than that during the non-plum rains season, which confirmed the correlation between allergens and plum rain climate. Further analysis showed that the positive rates of specific allergens such as mites, house dust, mold and animal dashes in the plum rains season were higher than those in the non-plum rains season, while the positive rates of tree pollen and plant pollen were opposite, which further confirmed that seasonal climate and environmental changes had a certain impact on the prevalence of allergens. In particular, the positive rate of mold in the plum rains season was significantly higher than that in the non-plum rains season. Therefore, the risk of atopic dermatitis among allergic people in high-temperature and high-humidity environments during the plum rains season in the Hunan area is significantly increased, and special attention should be paid to the prevention of atopic dermatitis caused by mold specific allergens.

This study also found that the prevalence rate in the male group was higher than that in the female group, which may be related to the physiological structure, lifestyle and hormone levels of the different genders. There are also significant differences in the positive rate of inhalation allergens in different age groups, and the positive rate of inhalation allergens in children is the highest. It may be that the regulation of the immune system in minors is not yet perfect, and the expansion of the range of activities after infancy increases the opportunity and variety of allergen exposure, leading to a greater possibility of allergic diseases. Therefore, more attention should be paid to allergic diseases in minors. Studies have reported that the prevalence of allergic diseases in male children in the Sichuan Province is much higher than that in female children.^[22]^ However, this phenomenon was not observed in this study, which may be due to the absence of gender differences in the incidence of atopic dermatitis in children.

Since the 1960s, IgE as a new indicator was introduced into the diagnosis of allergic diseases. Since then, specific IgE assays for different allergens have been gradually developed.^[23]^ At present, serum IgE allergen detection is relatively safe and stable, which can avoid possible severe allergic reactions in vivo experiments, and is considered to be the most effective in vitro allergen detection method.^[24]^ In this study, an enzyme-linked immunoassay method was used to detect the common aspiration allergen IgE antibodies in patients with atopic dermatitis in the Hunan area, and 94.19% of the total IgE-positive patients were found, indicating that the vast majority of patients could be well detected as hypersensitized. However, the positive rate for specific allergens was only 26.17%, and 68% of patients did not detect common specific allergens and could not be effectively treated. Currently, more than 20,000 types of allergens have been discovered, with more than 60 common types. A large number of newly discovered allergens have been discovered every year.^[17]^ Therefore, as found in this study, it is still difficult to identify allergens associated with allergic diseases. Therefore, discovery of new markers is imperative.

In recent years, routine blood inflammatory indicators, such as NLR, LMR, PLR and MPV/PC, have been increasingly reported in the literature on various diseases. KE et al found that the routine blood inflammatory biomarkers NLR and PLR are associated with increased all-cause and respiratory disease mortality in adult patients with asthma.^[25]^ Tarkowski et al also found that NLR and PLR could be used as inflammatory biomarkers for the efficacy of omalizumab in treating chronic treatment.^[26]^ This study also found that NLR and PLR were significantly increased and LMR was significantly decreased in the experimental group, indicating that routine blood inflammation indicators can also be used as potential markers for the diagnosis of atopic dermatitis. Interestingly, we also found that NLR, LMR and PLR levels in the IgE-negative group were also significantly different from those in the healthy group. Total negative allergens may be false negatives due to insufficient sensitivity of experimental detection, or it may be non-allergic dermatitis eczema caused by the release of inflammatory mediators caused by skin stimulation, such as endogenous atopic dermatitis with negative IgE, which is difficult to distinguish clinically. Therefore, there may also be a significant correlation between routine indicators of blood inflammation and IgE-negative atopic dermatitis in this study. There were no significant differences in NLR, LMR, PLR, or MPV/PC inflammatory indicators among the 3 groups in the experimental group, indicating that they could not distinguish subtypes of atopic dermatitis. ROC curve analysis showed that NLR had the highest diagnostic value in the diagnosis of atopic dermatitis, with an AUC of 0.725, sensitivity of 71.6%, and specificity of 62.3%. A cut-off value >1.405 may be used as a simple laboratory index for the clinical diagnosis of atopic dermatitis. Further analysis of atopic dermatitis in different subgroups showed that NLR had a higher value in the IgE single-positive group, with an area under the curve of 0.743. When the cut-off value of NLR was 1.405, the sensitivity and specificity reached 75.2% and 62.3%, respectively. Therefore, NLR may have better auxiliary diagnosis value for atopic dermatitis patients with single positive IgE.

Inhaled allergens are commonly used in the diagnosis of allergic diseases, and positive results only indicate the sensitization state of the body, which cannot accurately reflect the occurrence and development of allergic reactions. Moreover, 68% of the patients in this study did not have common specific allergens. In addition, there are too many types of allergens, and more screening is needed to identify specific allergens, which increases the financial burden on patients. As a calculation index in blood routine, NLR does not add additional examination costs to patients, and its price is much lower than that of mixed inhalation allergen test, NLR may have a greater predictive value over IgE indicators in patients with atopic dermatitis.

This study had some limitations. First, the data collected and analyzed in this study were from 2020 to 2022. Owing to the impact of the COVID-19 epidemic, the level of public health and awareness of self-protection have been enhanced, and the prevalence and spread of respiratory diseases other than COVID-19 have been controlled to some extent. Allergic diseases are also reduced to a certain extent by environmental changes.^[27]^ Similarly, the higher positive rates of inhaled allergens during the plum rains season and non-plum rains season were indoor allergens, which may be related to the reduction in outdoor activities caused by the COVID-19 epidemic. Second, this article only discusses the changes of allergens sensitization in the plum rains season and non-plum rains season from different time periods, which is easily affected by many factors and has certain limitations. Follow-up of patients to track the changes of allergens during different seasons will more directly reflect the impact of seasonal changes on individuals, which will be our next research direction. Third, neutrophil count, lymphocyte count, or NLR and other blood routine indicators are affected by factors such as age, gender and physical condition. Although we matched the different groups according to age, gender, health, etc, in order to exclude the influence of basic factors, we could not completely exclude the influence of potential confounding factors caused by other inflammatory diseases, which may lead to some bias. Fourth, the retrospective study only collected patient data without consulting doctors and other auxiliary means, which may lead to misdiagnosis.

## 5. Conclusions

In conclusion, the results of this study showed that dust mites and house dust were the most common inhaled allergens for atopic dermatitis in Hunan. The positive rate of inhaled allergens in males was higher than that in females, and the highest rate was found in children. The positive rate of inhaled allergens increased in plum rains season, especially mold allergens. NLR, LMR and PLR are correlated with atopic dermatitis, and NLR can be used as a potential marker for the diagnosis of atopic dermatitis.

## Author contributions

**Conceptualization:** Qian Sun.

**Data curation:** Qingyun Qu.

**Formal analysis:** Qingyun Qu.

**Investigation:** Qian Sun.

**Writing – original draft:** Qingyun Qu, Qian Sun.

**Writing – review & editing:** Qingyun Qu, Qian Sun.
